# Sorting secretory proteins

**DOI:** 10.7554/eLife.93490

**Published:** 2023-11-24

**Authors:** Anup Parchure, Julia von Blume

**Affiliations:** 1 Department of Cell Biology, Yale University School of Medicine New Haven United States

**Keywords:** trans-golgi network, secretory proteins, cell biology, TGN46, PAUF

## Abstract

A receptor protein called TGN46 has an important role in sorting secretory proteins into vesicles going to different destinations inside cells.

**Related research article** Lujan P, Garcia-Cabau C, Wakana Y, Lillo JV, Rodilla-Ramírez C, Malhotra V, Salvatella X, Garcia-Parajo MF, Campelo F. 2023. Sorting of secretory proteins at the trans-Golgi network by TGN46. *eLife* **12**:RP91708. doi: 10.7554/eLife.91708.

Approximately 14% of all protein-coding genes in humans produce proteins secreted by tissues and cells (Uhlén et al., 2019). These proteins control various essential processes, including immunity, metabolism and cellular communication. Secretory proteins also play significant roles in numerous diseases, such as neurological disorders and cancer (Brown et al., 2013).

Once a secretory protein has been synthesized in the endoplasmic reticulum, it must travel to another cellular compartment known as the trans-Golgi network before it can be released. The trans-Golgi network acts like a mail distribution centre, sorting secretory proteins into vesicles that travel to specific destinations, such as the cell surface, various compartments inside the cell (such as endosomes and lysosomes), or distinct domains in the plasma membrane (Figure 1A; Ford et al., 2021). A fundamental question in cell biology is how secretory proteins are effectively separated from one another and sorted into vesicles that will take the protein to the correct location.

**Figure 1.**
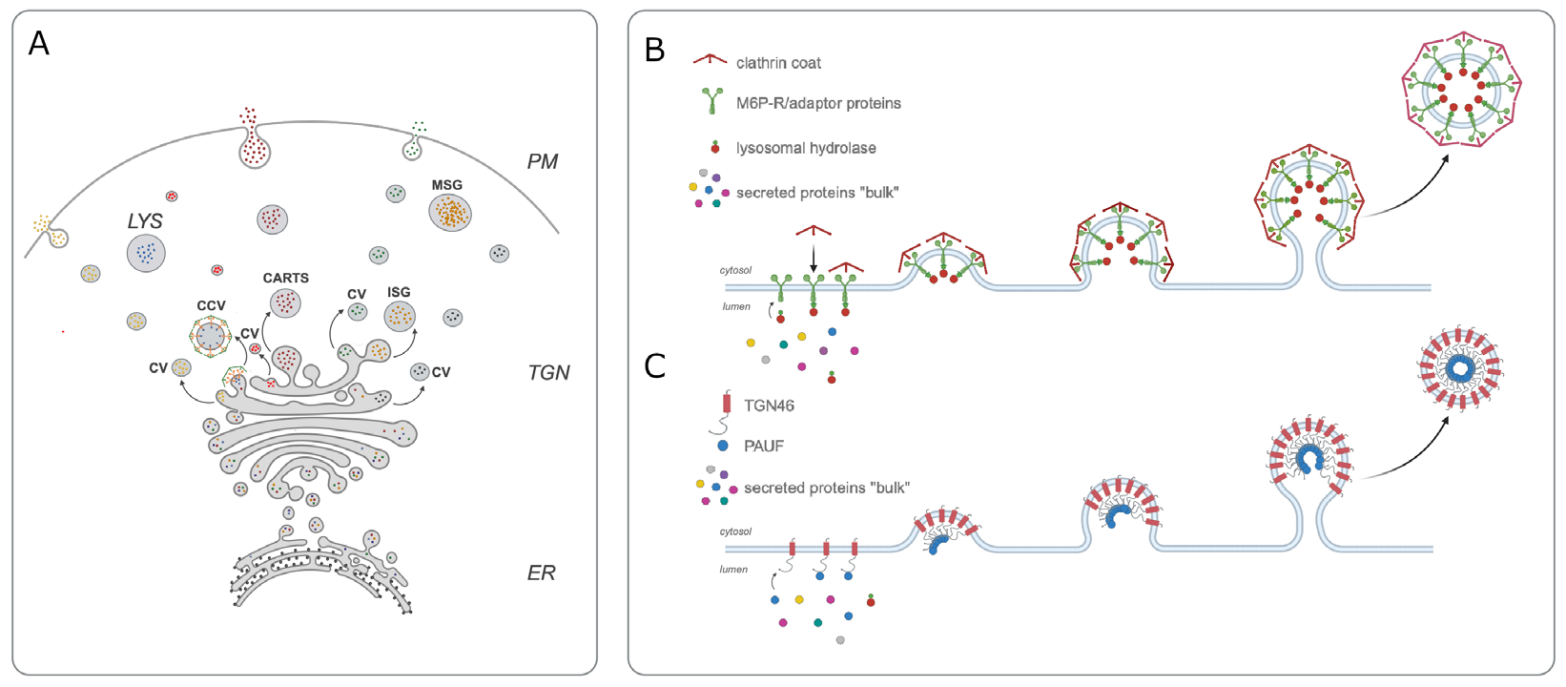
Cargo sorting at the trans-Golgi network. (**A**) In order for secretory proteins to reach their destination, they must first enter the endoplasmic reticulum (ER) and travel to the trans-Golgi network (TGN). Once there, proteins are sorted into different types of vesicles: constitutive vesicles (CV), clathrin-coated vesicles (CCV), the CARTS mentioned in the main text, or immature secretory granule (ISG). These vesicles carry a protein either to the plasma membrane (PM) to be immediately secreted, to mature secretory granules (MSG) to store it for future secretion, or to the lysosome (LYS) for degradation (**B**) Previous work showed that mannose-6-phosphate receptor (M6P-R, green) is responsible for sorting lysosomal enzymes (red small circles) into CCVs that are travelling to the lysosome. (**C**) Lujan et al. found that the transmembrane protein TGN46 (red) also acts as a cargo receptor and is responsible for sorting a secreted protein called PAUF (blue small circle) into CART vesicles destined for the cell surface. CARTS: CARriers TGN to the cell Surface; PAUF: pancreatic adenocarcinoma upregulated factor.

In the early 1980s, Stuart Kornfeld and colleagues provided the first mechanistic insight into protein sorting in the Golgi when they discovered a cargo receptor called M6P-R (short for mannose-6-phosphate receptor) within the membrane of the trans-Golgi network. This receptor can recognise and bind to a specific mannose-6-phosphate tag on lysosomal enzymes, and package them into vesicles destined for the lysosome (Dahms et al., 1989; Figure 1B).

This discovery raised the possibility that secretory proteins may be sorted by a similar receptor-ligand binding mechanism. However, the details of the sorting process remained a mystery. Now, in eLife, Felix Campelo (Barcelona Institute of Science and Technology) and colleagues – including Pablo Lujan as first author – report that a well-known protein called Transmembrane Network Protein 46 (TGN46) may be involved (Lujan et al., 2023).

TGN46 has long been hypothesized to carry out a sorting role in the trans-Golgi network as it displays many typical features for a cargo receptor. For instance, it rapidly cycles between the Golgi and plasma membrane, and the vesicles that transport it to the cell surface (known as CARTS) have been shown to carry secretory proteins, such as PAUF (short for pancreatic adenocarcinoma upregulated factor; Banting and Ponnambalam, 1997; McNamara et al., 2004). To investigate this hypothesis, Lujan et al. knocked out the gene for TGN46 from cells grown in a laboratory. This significantly reduced the amount of PAUF the cells secreted, suggesting that TGN46 acts as a cargo receptor for this secretory protein (Figure 1C).

Previous studies have shown that CARTS require an enzyme called protein kinase D in order to detach themselves from the membrane of the trans-Golgi network (Wakana et al., 2012). If this enzyme is inactivated and unable to perform this role, long tubules carrying cargo molecules will emerge from the Golgi membrane (Liljedahl et al., 2001). Lujan et al. found PAUF proteins in the tubules of wild-type cells but not in the tubules of the mutant cells lacking TGN46. This indicates that TGN46 is required to load PAUF into CART vesicles.

Next, Lujan et al. set out to find which parts of the TGN46 receptor were responsible for sorting and packaging secretory proteins. They found that the cytosolic domain of the receptor was not required for PAUF export, which is surprising given that adaptor proteins, which help to form vesicles, bind to other cargo receptors via this domain (Cross and Dodding, 2019). Changing the size of the transmembrane domain in TGN46 also did not impact the location of the receptor, as would be expected; instead it mildly affected how well PAUF was sorted into CARTS. Most importantly, Lujan et al. found that TGN46 only needs its luminal domain (the part of the receptor which faces the interior of the Golgi) in order to sufficiently sort PAUF proteins into CARTS.

This research sheds light on how secretory proteins are sorted into the correct vesicle at the trans-Golgi network. It also provides significant evidence that TGN46 acts as a cargo receptor, something which has been speculated in the field for over three decades. These exciting findings also raise new questions about how the luminal domain of TGN46 is able to recognize PAUF: is this recognition specific and based on ‘lock and key interactions’, or could it be based on the biophysical properties of the highly disordered luminal domain of TGN46?

It also remains to be seen which other secretory proteins TGN46 might be responsible for sorting. TGN46 is known to interact with another cell surface receptor called integrin beta 1, which is involved in cell adhesion at specific sites in the plasma membrane (Wang and Howell, 2000). This suggests that TGN46 may help to export proteins to specific domains at the cell surface. Future work investigating these questions, as well as others, will provide significant insights into the molecular mechanisms of protein secretion.
